# Facing fear through play: A smartphone-based proof-of-concept intervention using inverted augmented reality

**DOI:** 10.1016/j.invent.2026.100966

**Published:** 2026-06-23

**Authors:** Wolfgang Trapp, Susanne Röder, Franziska Wimmer

**Affiliations:** a Sozialstiftung Bamberg, Clinic for Psychiatry und Psychotherapy, Bamberg, Germany; b Department of General Psychology and Methodology, University of Bamberg, Germany

**Keywords:** Digital mental health, Exposure therapy, Gamification, Augmented reality, Oppositional action, Phobic disorders, Smartphone intervention

## Abstract

**Background:**

Despite the well-documented efficacy of exposure therapy for phobic disorders, its real-world implementation remains limited due to barriers in accessibility, acceptability, and generalization.

**Objective:**

This proof-of-concept study examined the feasibility and preliminary effects of a novel gamified augmented reality (AR) intervention designed to support in vivo exposure by embedding playful, non-threatening virtual stimuli into real-world anxiety-provoking contexts.

**Methods:**

Twenty individuals with various phobic disorders participated in a multiple-baseline design. At a randomly assigned time point, participants used a smartphone-based AR application, or in some cases, a VR headset version, to interact with virtual game elements within individualized fear-relevant environments. The task involved physically navigating individualized fear-relevant environments while collecting color-coded virtual objects presented via augmented reality within the real-world setting.

**Results:**

Linear mixed-effects analyses indicated a significant reduction in self-reported situational fear following the intervention, with a large within-subject effect size estimate (Cohen's d = 2.21). Secondary outcomes related to anxiety sensitivity, agoraphobic cognitions, and depressive symptoms also showed pre–post improvements with small to moderate effect sizes. No significant differences were observed across device types or treatment settings.

**Conclusion:**

The findings provide preliminary support for a low-threshold, smartphone-based AR approach that may complement exposure-based interventions in naturalistic settings. However, given the small and heterogeneous sample and the absence of a control group, these results should be interpreted cautiously. Future randomized controlled studies with larger and more homogeneous samples, validated behavioral outcome measures, and follow-up assessments are needed to clarify efficacy, mechanisms of change, and long-term effects.

## Highlights

• First AR exposure app to embed neutral elements into feared environments
• Gamified attention task was performed in vivo, promoting approach behavior
• Strong reduction in situational fear (primary outcome)
• Secondary improvements in anxiety and depression symptoms
• Low-cost smartphone design enables use in low-resource settings

## 1 Introduction

Phobic disorders are among the most common mental health conditions worldwide, affecting over 300 million people (GBD 2019 Mental Disorders Collaborators, 2022). While often considered less severe than other disorders, phobias can cause intense, uncontrollable fear that significantly impairs daily life, social participation, and overall well-being (Bandelow and Michaelis, 2015; Yang et al., 2021).

Exposure therapy, particularly in vivo exposure combined with cognitive restructuring, is the gold-standard treatment for phobias (Acarturk et al., 2009; Carpenter et al., 2018; Wolitzky-Taylor et al., 2008). Despite its strong evidence base, however, exposure therapy remains underutilized. Many individuals never seek treatment, and even among those who do, treatment refusal and dropout are common, particularly when exposure involves direct confrontation with feared stimuli (de Vries et al., 2021; Meyerbröker and Emmelkamp, 2010; Olatunji et al., 2009).

Recent theoretical developments have increasingly conceptualized exposure therapy within the framework of inhibitory learning (Craske et al., 2014). From this perspective, exposure is thought to facilitate the formation of new, non-threatening associations that inhibit, rather than erase, the original fear response. Repeated confrontation with feared stimuli across varying contexts may therefore strengthen inhibitory learning and promote greater flexibility in responding to fear-related cues.

At the same time, some authors have emphasized the potential importance of active engagement during exposure. Rather than merely enduring feared situations, individuals may benefit from actively performing goal-directed behaviors that counteract avoidance tendencies, sometimes referred to as “oppositional actions” or “threat-antagonistic actions” (Wolitzky and Telch, 2009)**.** For example, actively approaching a feared situation, such as running toward the railing of a high balcony, may introduce new, non-threatening associations into the fear structure while counteracting passive avoidance. Wolitzky and Telch (2009) further proposed that such “anti-phobic” actions may facilitate emotional processing during exposure and promote stronger reductions in fear across repeated exposure periods. By encouraging individuals to actively engage with feared situations instead of passively enduring them, such behaviors may additionally enhance perceived control and self-efficacy, both of which have been associated with improved anxiety outcomes (Paersch et al., 2025).

Digital interventions such as virtual reality (VR) and augmented reality (AR) have increasingly been explored as tools to support exposure-based interventions. VR-based exposure therapy has shown efficacy comparable to traditional in vivo exposure while offering greater control, flexibility, and accessibility (Carl et al., 2019; Morina et al., 2015; Wong et al., 2023). In contrast to VR, augmented reality interventions preserve the real-world environment while embedding virtual elements into naturalistic settings. Early AR-based exposure approaches demonstrated promising results particularly for specific phobias involving small animals (Botella et al., 2010, Botella et al., 2016). More recent work and systematic reviews further suggest that AR-based exposure interventions may represent a feasible and engaging extension of exposure therapy across a range of anxiety-related conditions (Hasan et al., 2023; Rajkumar, 2024).

More recently, researchers have begun to examine whether interactive and gamified task elements may further enhance engagement and adherence during exposure. For example, Lindner et al. (2020) and Zimmer et al. (2021) described automated and gamified VR- and AR-based exposure approaches for spider phobia that integrated playful task structures into the exposure process.

Building on these developments, the present approach was designed to combine real-world exposure with playful AR-based interaction. In contrast to conventional AR exposure approaches that superimpose feared virtual stimuli onto otherwise neutral environments, the intervention embeds non-threatening virtual game elements directly into fear-relevant real-world settings. Participants are encouraged to physically navigate and actively remain within the feared environment while interacting with AR-based targets, thereby potentially combining exposure-related “oppositional action” approach behavior with goal-directed playful engagement.

We suggest referring to this approach as Inverted Augmented Reality Exposure Therapy (iARET), reflecting the reversal of conventional AR exposure logic: rather than introducing feared virtual stimuli into safe environments, iARET introduces non-threatening virtual elements into feared real-world situations.

The aim of the present proof-of-concept study was to examine the feasibility and preliminary effects of iARET in individuals with phobic disorders using a multiple-baseline design. We expected that participants would show reductions in self-reported situational fear following the intervention. In addition, exploratory analyses examined changes in anxiety-related cognitions, anxiety sensitivity, and depressive symptoms, as well as potential differences across device types and treatment settings.

## 2 Methods

### 2.1 Design

This study employed a multiple-baseline design across participants with five assessment time points over five consecutive days. All participants completed a pre-assessment (T1) and a follow-up assessment (T5). Between these, three intermediate assessments (T2–T4) were conducted at daily intervals. The intervention—a smartphone-based augmented reality exposure game—was introduced at a randomly assigned time point (T2, T3, or T4), creating a staggered baseline structure across participants. This design was chosen to allow an initial examination of whether changes in situational fear occurred following the introduction of the intervention rather than merely over time, while maintaining flexibility for individualized exposure settings and minimizing participant burden.

Given the exploratory proof-of-concept character of the study, no formal a priori power analysis was conducted. The target sample size was determined pragmatically based on feasibility considerations and recruitment within the predefined study period.

### 2.2 Participants

A total of 20 individuals participated in the study (17 female, 3 male; M age = 24.7 years, SD = 8.7). No participants identified as non-binary or outside the gender binary.

Participants were recruited between February 12 and June 25, 2024. Recruitment took place via convenience sampling from three sources. Four participants were receiving inpatient treatment at the Department of Psychiatry and Psychotherapy, Sozialstiftung Bamberg. These individuals were considered sufficiently clinically stable to participate in the regular therapeutic program, including exposure-based interventions. Four additional participants were referred by outpatient psychotherapists who informed eligible patients about the study. Twelve participants were recruited via university mailing lists and flyers distributed at the University of Bamberg and the FHM University of Applied Sciences.

Interested individuals contacted the study team directly and underwent a clinical screening procedure conducted by a licensed clinical psychologist. During this screening, clinically relevant phobic symptoms and the primary feared situation were assessed based on ICD-10-oriented diagnostic criteria. For participants recruited from clinical settings, diagnoses were additionally supported by existing clinical records established by treating psychiatrists. Three interested individuals did not meet the criteria for clinically relevant phobic symptoms and were therefore not included in the study. Primary feared situations included spider phobia (*n* = 6), injection-related phobias (*n* = 4), and social phobia (n = 4). Additional feared situations included claustrophobia (*n* = 1), blood-related phobia (n = 1), snake phobia (n = 1), agoraphobia (n = 1), fear of choking while eating (n = 1), and fear of sharp edges (n = 1). No standardized structured diagnostic interview was administered specifically for the study.

Exclusion criteria comprised a diagnosis of schizophrenia or delusional disorder, electroconvulsive therapy (ECT) during or within one month prior to the study, and severe visual impairment interfering with the perception of AR content.

All participants provided written informed consent prior to enrollment. The study was approved by the ethics committee of the University of Bamberg (approval code: 2024–01/05; approval date: 22 January 2024) and conducted in accordance with the Declaration of Helsinki. The trial was preregistered in the German Clinical Trials Register (DRKS00033470) and is listed in the WHO International Clinical Trials Registry Platform.

### 2.3 Intervention

**Fig. 1:**
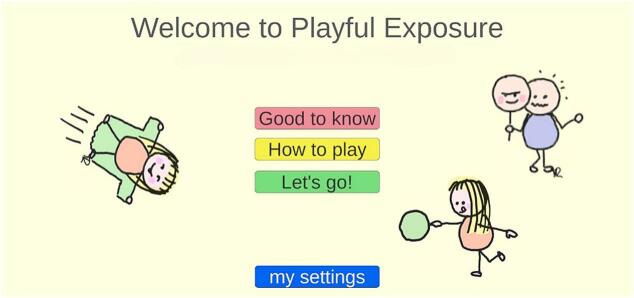
User interface of the augmented reality exposure app.

The intervention consisted of a gamified augmented reality (AR) application developed using Unity (version 2022.3.15f1, (2023c)) in combination with AR Foundation (version 5.1.2, (2023b)), Google's ARCore XR Plugin (version 5.1.2, (2023a)), and XR Plugin Management (version 4.4.1, (2023d)). The app was primarily deployed on Android smartphones. An early version was also implemented on the Oculus Quest 2 VR headset using passthrough mode. Fig. 1 shows the start screen of the application, which introduced participants to the task and allowed access to game instructions and settings.

Upon launching the application, participants saw this interface and were instructed to identify virtual spheres in a designated target color and “pop” them by physically approaching them. The spheres appeared spatially anchored in the users' real-world environment via augmented reality.

In the AR game, colorful virtual spheres were projected into the participant's real-world environment. Participants were instructed to identify and “pop” spheres of a designated target color by physically moving toward them and touching them with their smartphone. At any given time, only one sphere was visible. Its location was randomized across four cardinal directions (front, left, right, back), requiring users to scan and reorient themselves within the space. Spheres that did not match the target color disappeared automatically after a few seconds and were to be ignored. In contrast, target-colored spheres had to be actively “popped” through physical interaction. In the VR version, this was achieved by touching the sphere with a handheld controller while pressing a button.

**Fig. 2:**
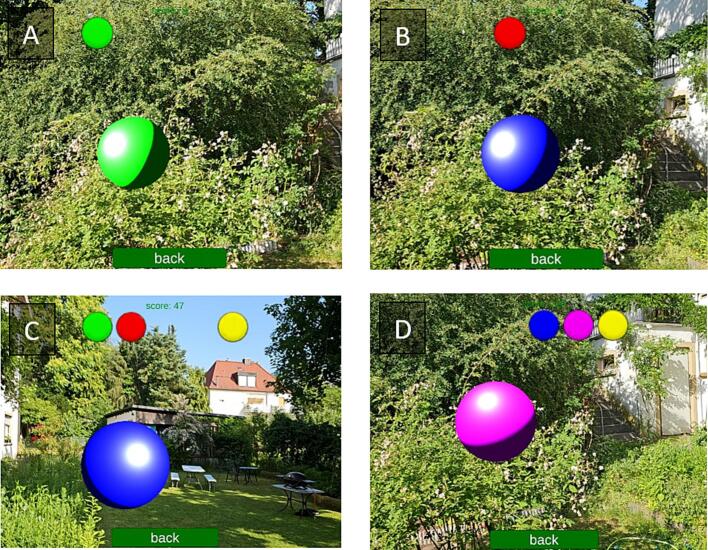
**Gameplay screenshots illustrating target selection and increasing difficulty in the AR exposure app.** Panels A and B depict early levels with a single target color. A: The green sphere should be popped, as it matches the target color. B: The blue sphere should be ignored, as the target is red. Panels C and D illustrate more advanced stages with multiple target colors and higher cognitive demand. C: The blue sphere is a distractor and should be ignored; valid targets are red, green, and yellow. D: The pink sphere should be popped, as pink is among the current targets.

Gameplay difficulty increased progressively across rounds. Modifications included shortened response times, the introduction of distractor colors, dynamic changes in the target color, and—at higher levels—the simultaneous presence of multiple target colors. Although the maximum difficulty level featured four target colors, the configuration with three targets proved most cognitively demanding. For that reason, three-target conditions were reserved for the final level of difficulty. An adaptive difficulty algorithm adjusted the game's complexity in real time: correct responses increased difficulty, while incorrect responses decreased it. Specifically, difficulty was recalibrated after every five spheres: if all five were responded to correctly—either by actively popping targets or ignoring non-targets—the difficulty level increased by one. If one or more errors occurred, the level decreased by one. Fig. 2 illustrates representative gameplay scenarios, including target selection and varying levels of cognitive demand.

Each round lasted 5 min, and participants could play up to three rounds. Before the first round, the experimenter demonstrated the game via screen mirroring on a large display. Self-reported anxiety ratings were collected before the first round and after each gameplay session. Depending on the individual exposure context and participant preference, participants completed between one and three gameplay rounds. Two rounds represented the most common procedure, whereas a third round could optionally be conducted when participants wished to continue or when fear levels remained elevated and further exposure was considered clinically appropriate. Consequently, participants contributed between two and four situational fear ratings on the intervention day, including the pre-intervention assessment. The total intervention time, including demonstration and up to three rounds, was approximately 20 min.

Exposure sessions took place in individualized fear-relevant real-world environments selected according to the participant's primary feared situation. Depending on the phobia, these settings included elevators, socially stressful situations, or other anxiety-provoking everyday contexts. For exposure tasks involving projected feared stimuli (e.g., spiders or snakes), dedicated rooms equipped with projection systems were available both at the Department of Psychiatry and Psychotherapy, Sozialstiftung Bamberg, and at the university setting. In cases of social anxiety, participants were asked, for example, to give a short presentation while simultaneously interacting with the AR task.

The experimenter accompanied participants during the intervention primarily to provide technical instructions, ensure safety, and collect anxiety ratings. No additional psychotherapeutic input or therapeutic guidance was provided during gameplay itself. Before the first round, participants received a brief demonstration of the game mechanics via screen mirroring on a large display.

Initially, the application was implemented using the Oculus Quest 2 in passthrough mode. After the first five participants, the protocol shifted to Android smartphones to improve ecological validity and accessibility. One participant with claustrophobia used the VR headset version, as mobile AR rendering proved unreliable in the mirrored elevator environment used for their exposure session.

### 2.4 Individualization of phobic contexts

To enhance ecological validity and maximize therapeutic relevance, the game-based intervention was embedded within real-world exposure contexts tailored to each participant's primary phobia. Participants engaged with the AR game while simultaneously being exposed to individualized fear-inducing stimuli or environments.

For participants with specific phobias such as arachnophobia or ophidiophobia, large high-resolution videos of spiders or snakes were projected onto opposing walls using video projectors. The AR game was configured so that virtual target spheres repeatedly appeared in close proximity to the projected stimuli, thereby encouraging approach behavior toward feared cues.

Participants with blood-injection-injury or surgical phobias were exposed to large-screen video content depicting relevant medical procedures. In cases of social phobia or agoraphobia, the intervention was conducted in vivo, for example during brief public speaking tasks or within busy public environments, depending on the participant's specific avoidance patterns.

### 2.5 Measures

#### 2.5.1 Primary outcome

The primary outcome was the subjectively perceived intensity of fear, assessed using a continuous visual analog scale (VAS) ranging from 0 (*no fear at all*) to 100 (*maximum imaginable fear*). Participants were asked: “How intense is your fear right now in the current situation?” Fear ratings were collected once daily across five consecutive days (T1–T5). On the intervention day (T2, T3, or T4, depending on group assignment), additional ratings were obtained after each gameplay session, resulting in up to four fear assessments on that day.

A VAS was selected because the individualized and heterogeneous nature of the phobic situations limited the applicability of a single disorder-specific questionnaire across participants. In addition, the repeated assessment of momentary situational fear during exposure contexts was considered particularly suitable for capturing short-term within-subject changes.

On assessment days without gameplay, participants were only briefly guided into their individualized fear-relevant situation to obtain situational fear ratings. After the fear rating had been collected, the exposure was terminated shortly thereafter and no extended exposure procedure was conducted on these occasions. In contrast, the intervention session involved approximately 20 min of sustained engagement with the AR-based gameplay task within the feared context. Thus, during four of the five assessment occasions, exposure to the feared situation was intentionally kept very brief and primarily served to assess situational fear rather than to induce meaningful exposure-related effects. The intervention session was the only occasion involving prolonged exposure within the feared context, thereby allowing exposure-related learning processes to occur in combination with the AR-based task.

#### 2.5.2 Secondary outcomes

To assess anxiety-related constructs and depressive symptomatology, participants completed a battery of standardized questionnaires before the first and after the final intervention session:

• The Body Sensations Questionnaire (BSQ; (Chambless et al., 1984) was used to assess fear of bodily sensations commonly associated with panic. The questionnaire consists of 17 items rated on a Likert-type scale, with higher scores indicating greater fear of anxiety-related bodily sensations. Example items include concerns about dizziness, heart palpitations, or shortness of breath. The BSQ has demonstrated good reliability and validity in anxiety-related samples.
• The Agoraphobic Cognitions Scale (ACS; (Chambless et al., 1984) was used to assess maladaptive and catastrophic thoughts associated with anxiety-provoking situations. Participants rate the frequency of thoughts such as “I might lose control” or “I might embarrass myself.” The ACS has shown good psychometric properties in both clinical and research settings.
• The Anxiety Sensitivity Index–3 (ASI-3; (Taylor et al., 2007) is an 18-item self-report measure assessing beliefs about the harmful consequences of anxiety symptoms across physical, cognitive, and social domains. Items are rated on a 5-point Likert scale ranging from 0 (“very little”) to 4 (“very much”). Example items include “It scares me when my heart beats rapidly.” The ASI-3 has demonstrated strong internal consistency and convergent validity.
• Depressive symptoms were assessed using the Beck Depression Inventory–II (BDI-II; (Beck et al., 2011), a widely used 21-item self-report questionnaire assessing depressive symptom severity over the past two weeks. Items are rated on a 4-point scale, with higher scores indicating more severe depressive symptoms. Example items assess sadness, guilt, or loss of interest. The BDI-II has demonstrated excellent reliability and validity across clinical and non-clinical populations.
• Additionally, the Montgomery–Åsberg Depression Rating Scale (MADRS; (Montgomery and Asberg, 1979) was administered as a clinician-rated measure of depressive symptom severity. The scale includes ten items assessing symptoms such as apparent sadness, inner tension, and reduced sleep. The MADRS has demonstrated good sensitivity to symptom change and strong psychometric properties.

Questionnaire data for the secondary outcome measures were collected in paper-and-pencil format. Participants completed the questionnaires one day before the first exposure session (T1) and again one day after the final exposure session (T5). Due to occasional missing questionnaire data, sample sizes varied slightly across secondary outcome analyses.

### 2.6 Statistical analysis

To examine the effects of the AR-based intervention on participants' fear ratings, we conducted a series of linear mixed-effects models using the nlme package (Pinheiro et al., 2024) in R version 4.4.1 (R Core Team, 2024). The dependent variable was participants' self-reported fear, rated on a visual analog scale from 0 to 100.

Each participant contributed four fear ratings:

1. The day before the intervention,
2. Immediately before the intervention session,
3. Immediately after the intervention session, and
4. The day after the intervention.

Based on these time points, two distinct within-subjects factors were constructed:

• Time (Before vs. After): comparing fear ratings before (points 1 and 2) with those after (points 3 and 4) the intervention;
• Exposure: comparing ratings during exposure (points 2 and 3) with those outside the exposure context (points 1 and 4).

Two between-subjects factors were also included:

• Device type: smartphone vs. VR headset;
• Treatment status: currently in inpatient vs. outpatient care.

Participants were modeled as random effects with random intercepts to account for inter-individual variability. Model building followed a stepwise approach: we started with a baseline model including random intercepts only. In subsequent steps, we added the main effect of Time, followed by the inclusion of Exposure, Device, and Treatment. Finally, we added interaction terms between Time and each of the other fixed effects. Model comparisons were conducted using likelihood-ratio tests. Assumptions (e.g., residual normality, homoscedasticity) were evaluated using diagnostic plots.

Given the small sample size, the mixed-effects models were specified parsimoniously and interpreted as exploratory. Linear mixed-effects modeling was chosen because it accounts for the repeated-measures structure of the data and the dependency of observations within participants. The primary inference focused on within-subject change over time, whereas analyses involving exposure context, device type, treatment status, and interaction terms were considered exploratory.

For secondary outcomes, paired-sample *t*-tests were conducted to assess changes from pre- to post-intervention on anxiety-related measures and depressive symptoms. Although depression was not a primary target, we expected potential improvements due to increased behavioral activation, perceived control, and self-efficacy. To account for multiple testing, Holm-adjusted *p* values were additionally calculated for the secondary outcomes.

To quantify within-subject changes in fear ratings, we calculated Cohen's d for paired samples using the effectsize package (Ben-Shachar et al., 2020). Confidence intervals were computed for all effect sizes.

## 3 Results

As shown in Table 1, participants reported high levels of fear both on the day prior to the intervention and immediately before the gameplay session.

**Table 1:** Mean self-reported fear ratings (0−100) across four time points during the intervention phase.

Time point	M (SD)
Day before exposure	75.1 (11.3)
Exposure day – before AR game	74.6 (11.9)
Exposure day – after AR game	44.2 (14.7)
Day after exposure	45.8 (17.6)

**Fig. 3:**
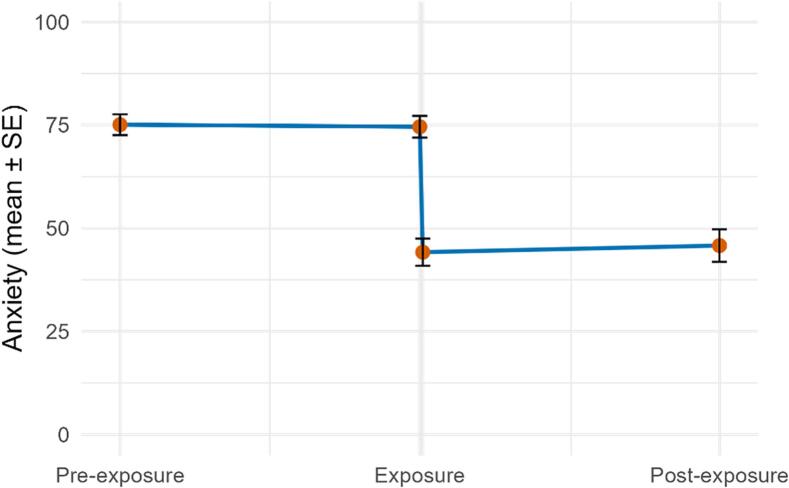
**Mean anxiety ratings (± standard error) across four assessment points.** The two time points on the intervention day (immediately before and after the AR task) are vertically aligned to illustrate the pronounced reduction in self-reported anxiety.

Following the AR-based intervention, subjective fear ratings dropped substantially and remained at a reduced level on the following day. Fig. 3 illustrates this trajectory, highlighting the steep decline in anxiety immediately after the intervention.

Linear mixed-effects modeling revealed a significant main effect of Time (pre vs. post), indicating a pronounced reduction in fear following the intervention, χ^2^(1) = 98.81, *p* < .001. None of the additional predictors—Exposure, Device type, or Treatment status—nor their interactions with Time significantly improved model fit (all χ^2^ < 4, all *p* > .27).

The effect size associated with the time-related change was large, *d* = 2.21, 95% CI [1.62, 2.78]. A detailed overview of the stepwise model comparison is provided in Table 2.

**Table 2:** Stepwise model comparison for linear mixed-effects analyses of fear ratings.

model	df	AIC	BIC	loglik	c^2^	∆df	p
Baseline model	5	717.55	729.46	−353.77	–	–	–
+ time	6	620.74	635.03	−304.37	98.81	1	<0.001
+ exposure, device, treatment	9	622.84	644.27	−302.42	3.91	3	0.272
+ interactions with time	12	625.06	653.65	−300.53	3.77	3	0.287

**Fig. 4:**
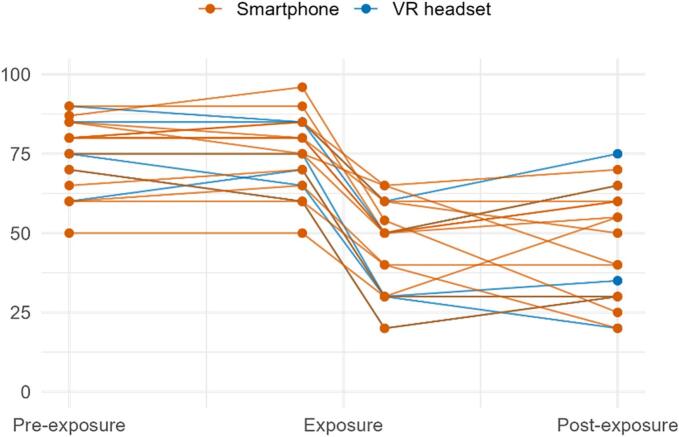
**Individual trajectories of anxiety ratings across the four assessment points, separated by device type.** Each line represents one participant (*N* = 20). The marked drop between the second and third measurement reflects the immediate effect of the AR-based intervention.

Fig. 4 displays individual fear trajectories separated by device type. Visual inspection confirmed a robust anxiety reduction for nearly all participants, with no notable differences between the smartphone and VR conditions.

**Fig. 5:**
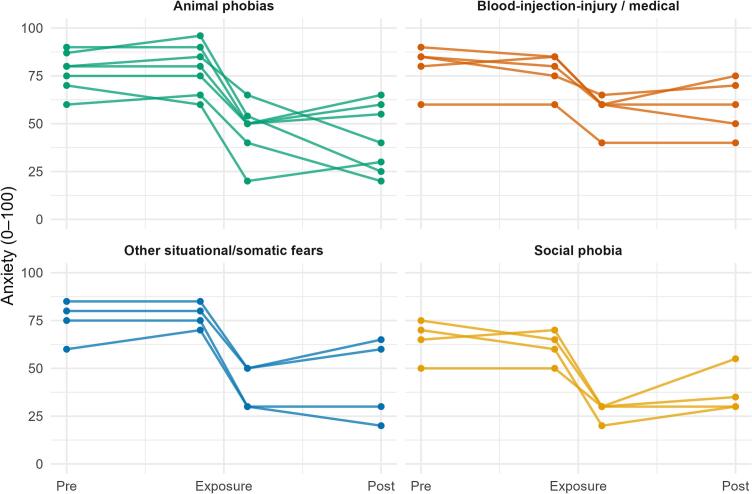
Exploratory individual fear trajectories grouped by broad phobia categories.

To provide additional descriptive insight into the heterogeneity of the sample, Fig. 5 displays exploratory individual fear trajectories grouped by broad phobia categories. Across categories, most participants showed marked reductions in situational fear between the pre- and post-exposure assessments, although substantial variability in baseline severity and response trajectories remained visible. Given the small subgroup sizes and exploratory nature of these visualizations, no formal subgroup comparisons were conducted.

Panels display descriptive trajectories for participants with animal phobias, blood-injection-injury/medical phobias, social phobia, and other situational/somatic fears. Categories were collapsed descriptively due to the small number of participants per specific phobia type and should not be interpreted as formal subgroup comparisons.

In addition to the primary outcome, paired-sample *t*-tests revealed significant improvements on several secondary measures, including anxiety sensitivity, agoraphobic cognitions, and depressive symptoms. A full overview of all pre–post comparisons is provided in Table 3.

**Table 3:** Results of paired-sample t-tests and effect sizes (Cohen's d) for all secondary outcome measures.

Measure	Pre (*M* ± *SD*)	Post (*M* ± *SD*)	t(df)	*p*	*p*Holm- adjusted	Cohen's *d* [95% *CI*]
BSQ	2.45 ± 0.74	2.22 ± 0.77	1.79(16)	0.093	0.093	0.43 [−0.07, 0.93]
ASI-3	38.5 ± 11.6	32.8 ± 10.3	3.28(18)	0.004	0.020	0.75 [0.23, 1.26]
ACS – Frequency	1.88 ± 0.61	1.58 ± 0.48	3.35(18)	0.004	0.020	0.77 [0.25, 1.27]
ACS – Probability	22.4 ± 15.7	16.6 ± 12.9	2.50(18)	0.022	0.044	0.57 [0.08, 1.05]
BDI-II	14.0 ± 12.4	10.8 ± 11.6	3.03(18)	0.007	0.021	0.70 [0.18, 1.19]
MADRS	9.1 ± 8.21	6.15 ± 5.70	3.41(19)	0.003	0.018	0.76 [0.25, 1.25]

*Note. M* = Mean; *SD* = Standard Deviation; *p*Holm = Holm-adjusted *p* values; *CI* = Confidence Interval; *p*-values based on two-tailed tests. BDI-II = Beck Depression Inventory II; MADRS = Montgomery-Åsberg Depression Rating Scale; BSQ = Body Sensations Questionnaire; ASI-3 = Anxiety Sensitivity Index-3; ACS = Agoraphobic Cognitions Scale.

Scores on the Anxiety Sensitivity Index-3 and both subscales of the Agoraphobic Cognitions Scale showed significant pre–post improvements, with medium to large effect sizes. Although scores on the Body Sensations Questionnaire also decreased, this effect did not reach statistical significance. Significant reductions were observed in both self-reported (BDI-II) and clinician-rated (MADRS) depressive symptoms.

Taken together, these findings suggest substantial reductions in situational phobic fear, accompanied by small to moderate improvements in anxiety-related and depressive symptoms.

## 4 Discussion

### 4.1 Main findings and interpretation

This study evaluated a novel smartphone-based augmented reality (AR) intervention designed to support in vivo exposure in individuals with phobic disorders. Participants showed marked reductions in self-reported situational fear across the intervention period, accompanied by a large within-subject effect size estimate (Cohen's d = 2.21). Given the exploratory proof-of-concept design, the absence of a control group, and the small heterogeneous sample, these findings should be interpreted cautiously. Nevertheless, the observed reductions in situational fear suggest that combining exposure-related approach behavior with playful AR-based interaction may represent a promising direction for future exposure-based interventions.

The intervention appeared feasible and acceptable across the different exposure settings. Most participants completed the planned exposure sessions, and several informally expressed that the playful task structure helped them remain engaged during exposure. No significant effects emerged for device type, exposure timing, or treatment status. However, these subgroup-related findings remain preliminary given the small and unbalanced sample, which limited statistical power to detect potential differences between subgroups.

The observed reductions in fear may reflect several mechanisms discussed in the theoretical framework of the present study, including inhibitory learning processes and the effects of active, exposure-related approach behavior. By combining real-world exposure with playful and goal-directed interaction, the intervention may have helped participants remain engaged with feared situations while encouraging repeated approach rather than avoidance. In addition, the interactive nature of the task may have contributed to perceived control and self-efficacy during exposure (Paersch et al., 2025; Ulleberg et al., 2021).

### 4.2 Secondary outcomes

In addition to situational fear, participants also showed significant pre–post improvements in anxiety-related beliefs (ASI-3, ACS) and depressive symptoms (BDI-II, MADRS). These secondary outcomes were not the primary target of the intervention but are theoretically plausible. The intervention required participants to actively approach and interact with fear-related environments, potentially challenging maladaptive cognitions related to avoidance, helplessness, and vulnerability (Beck, 1980; Beck and Emery, 2005). As discussed in the previous section, repeated engagement with feared situations through playful and goal-directed interaction may also have contributed to increased perceived control and self-efficacy, both of which are known to be associated with reduced anxiety and depressive symptoms. This may help explain why the intervention was accompanied by broader improvements in cognitive and affective functioning observed in the present study.

However, effect sizes for these secondary outcomes (d = 0.70–0.77) were notably smaller than the primary effect (d = 2.21), indicating that the intervention's strongest impact was on situational fear. At the same time, nonspecific factors such as expectancy effects, novelty, or experimenter support may also have contributed to the observed changes.

### 4.3 Limitations

Several limitations should be acknowledged. First, the study employed a single-group pre–post design without follow-up assessments, precluding conclusions about long-term efficacy, maintenance of treatment gains, and causal mechanisms. Although both traditional exposure-based interventions (Carpenter et al., 2018) and earlier AR-based exposure approaches (Botella et al., 2010) have shown relatively stable effects over time, it remains unclear whether the present gamified AR-based intervention would produce durable improvements beyond the immediate intervention period. In particular, short-term reductions in situational fear may partly reflect expectancy effects, novelty-related engagement, or temporary motivational activation associated with the playful intervention format. These factors may potentially produce less stable effects than those typically observed in more established exposure-based treatments. Future randomized controlled studies with longer follow-up periods are therefore necessary to examine the sustainability and generalizability of the observed effects.

Second, the present multiple-baseline design included only a limited number of assessment points over a relatively short time period. Although the staggered introduction of the intervention allowed a preliminary examination of whether reductions in situational fear occurred following the introduction of the intervention, the small number of repeated measurements limited the ability to model more complex temporal dynamics and individual response trajectories. Accordingly, while linear mixed-effects models accounted for inter-individual variability, we did not include random slopes or autoregressive error structures due to the small sample size, limited statistical power, and limited number of observations per participant. Future studies should employ more extended longitudinal designs with additional assessment points to allow more fine-grained modeling of change processes over time.

Furthermore, randomized controlled trials will be necessary to strengthen causal inferences and help disentangle specific intervention effects from nonspecific factors such as expectancy, novelty, or experimenter support. In line with recent recommendations for comparator selection in digital mental health research (Goldberg et al., 2023), future studies should additionally examine which control conditions are most suitable for isolating the specific contribution of the AR-based gameplay component. Potential comparators may include standard exposure procedures, exposure combined with non-gamified smartphone interaction, or exposure tasks involving attentional engagement or motor activity without active AR-based approach behavior. Such designs may help disentangle the relative contributions of exposure itself, attentional engagement, motor activity, oppositional approach behavior, and AR-specific novelty effects.

The large within-subject effect size observed in the present study should be interpreted cautiously. Effect size estimates derived from uncontrolled within-subject designs may overestimate treatment-specific effects relative to between-group comparisons in randomized controlled trials, particularly when expectancy effects, nonspecific engagement effects, or regression to the mean cannot be ruled out.

The primary outcome measure focused on subjective situational fear assessed via a visual analog scale. Although this approach allowed repeated real-time assessment across highly individualized exposure settings and heterogeneous phobia types, it does not capture broader aspects of clinical severity, avoidance behavior, or functional impairment. Single-item VAS ratings are inherently context-dependent and may be influenced by situational or expectancy-related factors. The highly individualized exposure contexts additionally complicate the interpretation of the observed effects, as exposure situations differed substantially with regard to controllability, duration, and intensity across participants. Consequently, it remains difficult to disentangle the relative contributions of the AR-based gameplay component, the exposure itself, and other contextual influences.

The absence of a standardized behavioral approach test (BAT) represents an additional limitation. Such procedures were difficult to implement consistently across the heterogeneous phobia types and individualized exposure contexts included in the present study. Future research may therefore benefit from incorporating more individualized behavioral outcome measures tailored to specific fear contexts, such as approach distance, time spent in feared situations, or observable avoidance behavior. At the same time, highly individualized behavioral assessments would introduce additional heterogeneity across outcome measures, potentially complicating comparability across participants and phobia types. The present proof-of-concept study therefore prioritized a transdiagnostic assessment approach that could be applied consistently across diverse fear contexts. In addition, repeated real-world assessments delivered via smartphones or ecological momentary assessment methods may help capture how fear, avoidance, and engagement fluctuate across everyday situations and over longer time periods.

Finally, the sample was relatively young and predominantly female, with many participants being university students who were not currently receiving treatment. These characteristics may limit the generalizability of the findings to more chronic or clinically severe populations. Future research should therefore aim for more diverse and clinically representative samples and explore potential differences across demographic and diagnostic subgroups.

### 4.4 Clinical and digital health implications

The intervention evaluated in this study exemplifies a promising innovation in digital exposure therapy—one that is mobile, gamified, and embedded in the user's real-world environment. By leveraging augmented reality on everyday smartphones, it avoids many logistical barriers associated with traditional in vivo or virtual reality exposure, while retaining core elements of exposure-based interventions. This low-cost, broadly accessible format may be particularly suitable for settings with limited mental health infrastructure or personnel.

The use of inverted augmented reality, in which neutral stimuli are embedded into personally feared environments, introduces a novel therapeutic principle: Rather than relying on virtual representations of feared stimuli, the intervention promotes real-world exposure via playful interaction. This design may help bypass avoidance tendencies and foster a sense of control, thereby enhancing motivation and adherence—two well-known challenges in traditional exposure therapy (Fleming et al., 2017; Johnson et al., 2016).

Moreover, the gamified structure invites repeated use, potentially supporting the generalization and maintenance of therapeutic effects beyond clinical sessions. Given the well-documented difficulties in promoting long-term generalization in exposure therapy (Craske et al., 2014; Swan et al., 2016), this is a particularly promising feature. At the same time, the present approach also raises theoretically important questions regarding attentional processes during exposure. Inhibitory learning models emphasize the importance of expectancy violation and sustained engagement with feared stimuli during exposure-based interventions (Craske et al., 2014). From this perspective, it remains unclear to what extent the gamified attentional demands of the AR task may facilitate approach behavior and emotional engagement versus partially functioning as a form of distraction. Future studies should therefore examine more directly how attentional allocation during AR-supported exposure influences inhibitory learning processes and long-term treatment outcomes.

In line with digital health objectives, the app can function either as a stand-alone intervention or be integrated into stepped-care or blended treatment models, increasing flexibility and scalability. Furthermore, the core task mechanics could be adapted for use in immersive VR, 360° video, or web-based platforms, making the intervention compatible with a wide range of technological infrastructures and clinical delivery formats.

## 5 Conclusion

In sum, the present proof-of-concept study provides preliminary evidence that a gamified, smartphone-based augmented reality intervention may help reduce situational fear in individuals with heterogeneous phobic symptoms. The findings further suggest that integrating playful AR-based interaction into real-world fear contexts represents a potentially promising direction for digital exposure-based interventions.

Crucially, the intervention introduces a novel therapeutic principle: instead of superimposing phobia-related stimuli onto neutral contexts, as in many traditional AR-based approaches, it embeds neutral yet playfully engaging game elements into fear-evoking real-world environments, thereby inverting the typical AR logic. This design aims to encourage real-life exposure through playful interaction and may offer a flexible and scalable approach for increasing engagement within exposure-based interventions.

At the same time, the present findings remain preliminary given the small heterogeneous sample, the uncontrolled design, the reliance on self-reported fear ratings, and the absence of long-term follow-up assessments. Future studies should therefore employ randomized controlled designs, include behavioral and disorder-specific outcome measures, examine mechanisms of change more directly, and evaluate longer-term treatment outcomes across more diverse clinical populations.

## Abbreviations

**ACS** – Anxiety Control Scale; self-report measure assessing perceived control over anxiety-related events.

**AR** – Augmented Reality; technology that overlays digital elements onto the real-world environment.

**ASI-3** – Anxiety Sensitivity Index, version 3; measures fear of anxiety-related sensations.

**BDI-II** – Beck Depression Inventory, Second Edition; self-report questionnaire assessing depressive symptoms.

**BSQ** – Body Sensations Questionnaire; assesses fear of bodily sensations often associated with panic.

**LMM** – Linear Mixed-Effects Model; a statistical method for analyzing repeated-measures data.

**MADRS** – Montgomery-Åsberg Depression Rating Scale; clinician-rated scale for evaluating depression severity.

**VR** – Virtual Reality; immersive technology that simulates environments through head-mounted displays.

## Declaration of Generative AI and AI-assisted technologies in the writing process

The authors acknowledge the use of generative AI (ChatGPT by OpenAI) in the preparation of this manuscript. The AI tool was used to assist in drafting, structuring, and refining specific sections of the text. The authors reviewed and edited all content to ensure accuracy and take full responsibility for the final version.

## Funding sources

This research did not receive any specific grant from funding agencies in the public, commercial, or not-for-profit sectors.

## Declaration of competing interest

The authors declare that they have no known competing financial interests or personal relationships that could have appeared to influence the work reported in this paper.

## Data Availability

The dataset analyzed during the current study is not publicly available due to data protection and ethical considerations. De-identified data may be made available from the corresponding author upon reasonable request.
